# Comprehensive gene panels provide advantages over clinical exome sequencing for Mendelian diseases

**DOI:** 10.1186/s13059-015-0693-2

**Published:** 2015-06-26

**Authors:** Saudi Mendeliome Group

**Affiliations:** Saudi Human Genome Project, King Abdulaziz City for Science and Technology, Riyadh, Saudi Arabia

## Abstract

**Background:**

To understand the contribution of Mendelian mutations to the burden of undiagnosed diseases that are suspected to be genetic in origin, we developed a next-generation sequencing-based multiplexing assay that encompasses the ~3000 known Mendelian genes. This assay, which we term the Mendeliome, comprises 13 gene panels based on clinical themes, covering the spectrum of pediatric and adult clinical genetic medicine. We explore how these panels compare with clinical whole exome sequencing (WES).

**Results:**

We tested 2357 patients referred with suspected genetic diagnoses from virtually every medical specialty. A likely causal mutation was identified in 1018 patients, with an overall clinical sensitivity of 43 %, comparing favorably with WES. Furthermore, the cost of clinical-grade WES is high (typically more than 4500 US dollars), whereas the cost of running a sample on one of our panels is around 75–150 US dollars, depending on the panel. Of the “negative” cases, 11 % were subsequently found by WES to harbor a likely causal mutation in a known disease gene (largely in genes identified after the design of our assay), as inferred from a representative sample of 178. Although our study population is enriched for consanguinity, 245 (24 %) of solved cases were autosomal dominant and 35 (4 %) were X-linked, suggesting that our assay is also applicable to outbred populations.

**Conclusions:**

Despite missing a significant number of cases, the current version of the Mendeliome assay can account for a large proportion of suspected genetic disorders, and provides significant practical advantages over clinical WES.

**Electronic supplementary material:**

The online version of this article (doi:10.1186/s13059-015-0693-2) contains supplementary material, which is available to authorized users.

## Background

The contribution of genetic variation to human diseases is an old concept, but its realization in the practice of medicine has been challenging. For most diseases, the genetic risk is both modest and strongly influenced by environmental risk factors such that the medical actionability of the identified genetic risk is limited [1, 2]. On the other hand, there are diseases that are traceable to mutations in single genes. These so-called Mendelian diseases offer a unique window into the physiological role of individual genes across tissues and stages of development [3]. From a clinical perspective, Mendelian mutations are the most medically actionable genetic variants in that they can be assigned a causal role with the attendant management and prevention benefits.

Although the first Mendelian mutation was identified in the 1980s, less than 200 Mendelian genes were known by the turn of the 21st century [4]. The publication of the human genome and the development of new analytical tools to study it, primarily next-generation sequencing (NGS), have sparked an unprecedented race to identify the suspected thousands of Mendelian genes, and as of the writing of this manuscript, more than 3000 genes are listed in the Online Mendelian Inheritance in Man (OMIM) database as disease causing [5–7]. With the discovery of each Mendelian gene, there is the immediate translational benefit of offering a precise molecular diagnosis for more patients and their families.

In order to reap the benefits of this growing knowledge of genes that cause human diseases in a Mendelian fashion, genome sequencing tools have quickly been introduced into the realm of clinical diagnostics [8]. While sequencing of the entire genome represents the most comprehensive option, sequencing of its ~2 % coding part (whole-exome sequencing [WES]) has emerged as a cheaper and more practical alternative [9]. Application of WES in the clinic to explain the etiology of suspected Mendelian disorders has been relatively successful. A few large studies on the clinical utility of WES on a range of disorders, mostly neurological, have reported a yield of around one in four, making it the highest yield test yet in the clinical geneticist’s armamentarium [10–12].

The cost of clinical-grade WES is high, typically > US$4500 (higher when done as a child–parents trio), with a long turnaround time of three months on average. These drawbacks are primarily driven by the interpretation challenge. WES typically uncovers numerous variants, and identifying the one causal variant can be a challenge. Despite the development of several tools that aid in the automation of prioritizing WES variants, the requirement for manual inspection and expert analysis remains. In addition, there is a growing concern about the potential of WES to reveal medically actionable results that are unrelated to the original indication for the test (secondary or incidental findings) and how best to communicate them to patients [13].

An alternative approach to WES is gene panels in which an assortment of genes deemed relevant to a particular phenotype are sequenced in patients with that phenotype. Such a focused analysis of a subset of clinically relevant genes obviates or minimizes some of the key challenges described above for WES [14, 15]. One key difficulty in the design of these panels is defining the appropriate list of genes for a given phenotype owing to the remarkable variability of clinical presentations. Indeed, gene panels developed by different labs for the same indication are seldom identical and the discrepancy can be significant [16]. In this study, we took a different approach whereby we relaxed the clinical indication such that minimal clinical expertise is required to order the appropriate panel; e.g., unusual facial appearance will trigger testing for the Dysmorphology/Dysplasia panel, whereas acidosis will trigger testing for the Inborn Errors of Metabolism panel. This very inclusive design allowed us to define 13 very broad clinical themes among which a set of ~3000 Mendelian genes (as of August 2013 when the assay was designed) were distributed. We describe the successful testing of this assay on more than 2300 patients with diagnoses spanning the spectrum of medical and surgical specialties. We implemented a multiplexing strategy to run more than one sample on the high throughput sequencer. This contributed to the reduction of the sequencing cost, which ultimately reduced the final sample processing cost.

## Results

### High analytical sensitivity and specificity of the Mendeliome assay

We used 642 samples with known mutations to calculate the analytical sensitivity of the Mendeliome assay. Overall analytical sensitivity was 79 % (507/642). The Mendeliome assay missed 135 known mutations, 46 % (62/135) of which were due to a design flaw, i.e., the disease gene was not included in the panel appropriate for the disease presentation. If these 62 cases were excluded (genes can easily be added to the panel, see below), the overall analytical sensitivity would increase to 87 % (507/580). Based on these positive controls (580), sensitivity for single nucleotide variants was found to be 93 % (398/428). However, sensitivity for indels was lower at 72 % (109/152). As expected for the semiconductor-based ion torrent sequencing, the bias against indels was not uniform but was largely sequence context-dependent, especially around homopolymer regions [17]. In addition to these positive controls, we used single nucleotide polymorphism (SNP) genotyping arrays (Affymetrix Axiom GT1 chip with ~580,000 SNPs) coming from 21 patients as a second method of testing the analytical sensitivity. We compared the variants detected by SNP arrays with those detected by the NGS technology for each sample. From a total of 3319 SNPs lying within our target regions of the panels, the resulting SNP sensitivity was about 95 %. Interestingly, we identified 30 extra SNPs that were called by the assay but were not called with high confidence on the chip. For analytical specificity, we used a predetermined quality score of 100 (this takes into account strand bias, homopolymer errors, among others; see “Materials and methods”). Analytical specificity was based on the Sanger validation of 1078 variants called by the assay. Sanger sequencing confirmed 93 % (819/881) of single-nucleotide variants and 78 % (154/197) of indels that met or were higher than that quality score.

### High yield of the Mendeliome assay in the clinic

A total of 2357 patients representing a very wide range of suspected genetic diseases were tested by the Mendeliome assay (see Table 1 for the number of patients tested on each panel). Only one panel was chosen per patient based on the most prominent “primary clinical feature” (see “Materials and methods”). The overall clinical sensitivity — i.e., detection of a likely causal variant that is subsequently confirmed by Sanger sequencing — was 43 %. Table 1 also summarizes the clinical sensitivity per panel as well as per clinical feature within each panel. As expected, specialties with the highest referral rate were neurology, dysmorphology, pediatric ophthalmology and immunology because of the nonspecificity of the clinical presentation, extreme and genetic heterogeneity, and because a genetic cause is highly suspected for a large fraction of their patient populations. Indeed, we note a relatively high yield for the respective panels of 40 %, 38 %, 52 %, and 37 % (Table 1). Specificity of the presentation appeared to bear appreciably on the clinical sensitivity of the assay. For example, with an objective evidence of skeletal dysplasia the sensitivity of the Dymorphology/Dysplasia panel was 45 % compared with 32 % when any degree of dysmorphism was used as the entry point. Similarly, the finding of a specific pattern of neurological abnormality (e.g., muscular dystrophy and neurodegenerative disorders) was associated with a much higher sensitivity compared with non-syndromic developmental delay/intellectual disability of any degree (56 % and 42 % versus 11 %). Also consistent with this is the finding that retinal dystrophies (almost always Mendelian in etiology) were more likely to have positive hits than the overall performance of the Vision panel (65 % versus 52 %).

**Table 1:** Clinical sensitivities

Gene panel type	Total patients run	Overall clinical sensitivity	Selected subgroup clinical sensitivity	
Cardiovascular	243	28 %	Cardiomyopathy	32 %
			Congenital heart disease	10 %
			Arrhythmias	31 %
			Aneurysms	29 %
Deafness	147	54 %	Hearing loss	-
Dermatology	68	62 %	Various dermatological features	-
Dysmorphology-Dysplasia	354	38 %	Skeletal dysplasia	45 %
			Dysmorphism	32 %
Endocrinology	36	61 %	Pituitary and thyroid disorders	-
Gastroenterology	73	29 %	Persistent jaundice	-
Hematology	33	24 %	Aplastic anemia	-
Inborn errors of metabolism	122	59 %	Metabolic disorders	-
Neurology	524	40 %	Syndromic DD/ID (recognizable syndromes)	47 %
			Syndromic DD/ID NYD (not yet determined, unrecognizable syndrome)	25 %
			Structural brain (cerebral/cerebellar/brain stem) and spinal malformations/anomalies^a^	34 %
			Non-syndromic DD/ID NYD (not yet determined, unrecognizable)^b^	11 %
			Neurodegenerative disorders	42 %
			Coordination^c^/movement disorders	69 %
			Peripheral neuropathy	33 %
			Myopathies/joint abnormalities^d^	56 %
PID	196	37 %	Primary immunodeficiency disorders	-
Pulmonology	36	36 %	Chronic lung infection suspected cystic fibrosis	-
Renal	107	57 %	Glomerular/tubular disorders	41 %
			Cystic kidney disease	63 %
			Kidney malformation	69 %
Vision	418	52 %	Retinal dystrophy (syndromic, non-syndromic, RP, CRD, macular dystrophy, FEVR, GFS)	65 %
			Cataract (syndromic and non-syndromic)	34 %
			Aniridia	33 %
			Microphthalmia/anophthalmia (with and without coloboma)	30 %
			Corneal dystrophy (CHED and all other subtypes)	40 %
			Others	23 %

^a^Primary microcephaly cases are included in this group

^b^Non-syndromic cases of autism/mental disorder and epilepsy are included under this group

^c^Ataxia cases secondary to cerebellar hypoplasia are included under the structural brain abnormalities group

^d^Cases with arthrogryposis multiplex syndromes are included under the myopathies group

*CHED* corneal hereditary endothelial dystrophy, *CRD* cone-rod dystrophy, *DD* developmental delay, *FEVR* familial exudative vitreoretinopathy, *GFS* Goldmann-Favre syndrome, *ID* intellectual disability, *NYD* not yet determined, *PID* primary immunodeficiency, *RP* retinitis pigmentosa

### The Mendeliome assay performs favorably compared with WES

The clinical sensitivity of our assay (43 %) is comparable to the ~25 % reported by several large clinical WES studies [10–12]. The Mendeliome assay is inherently limited to established disease genes, so it will miss cases caused by large structural variants and mutations in novel genes, although the design is flexible and allows for the addition of newly published disease genes as frequently as needed, e.g., every six months. We have queried OMIM on 30 March 2015 and found <170 OMIM genes that are not in the current design of the assay but are eligible for inclusion due to high quality of the disease link. These can be spiked into the existing two tube multiplex PCR. Even if we have to introduce these additional genes as an additional PCR (rather than spike in) for each panel, the cost of that additional PCR will be < $10 since the product will be pooled with the product of the other two tube multiplex PCR. Nonetheless, in order to assess the magnitude of this limitation, we randomly selected 213 cases that are negative by the Mendeliome assay and processed them using molecular karyotyping. Thirty-five of these were found to have likely pathogenic de novo copy number variations (CNVs) [18]. If we were to exclude these 35 cases, our clinical sensitivity would increase slightly to 44 %. The remaining 178 were processed using WES, and only 11 % (20/178) were found by WES to have a mutation in a known gene that was missed by the Mendeliome assay. Out of these 20 missed cases, the majority (*n* = 14, 70 %) were due to a design flaw, i.e., the disease gene was not included in the panel appropriate for the disease presentation, and this can easily be fixed by the spike-in strategy mentioned above. The remaining six cases represent limitation of the analytical sensitivity of the NGS platform we used in this study. On the other hand, we note that our cohort included two patients who had had negative diagnostic WES results prior to their enrollment in the Mendeliome assay, and were found to have likely causal mutations by the latter. These cases were missed at the interpretation phase of WES analysis and were solved by the Mendeliome assay likely because of the smaller number of variants. The much smaller number of variants to be queried by the Mendeliome assay versus WES also meant a much more rapid clinical interpretation (average 20 min per panel versus 2–3 hours per WES). This has markedly reduced the cost of interpretation on top of an already appreciable reduction in running cost (24 panel samples were run per chip versus one WES per chip). The cost is estimated to be $150 per sample with a range of $75–150 per sample depending on the panel selected. Also relevant to cost reduction is that we have had five couples who lost children with a likely recessive disease but we had no access to DNA from the deceased children. By running the appropriate panel on both parents we were able to identify the likely causal mutation at a much lower cost than the duo WES design that would have been required to reach the same conclusion. Thirty-one de novo mutations were identifiable as likely disease-causing heterozygous mutations in relevant Mendelian genes, and their de novo status was confirmed by Sanger sequencing of a single amplicon in both parents.

### The Mendeliome assay expands the clinical spectrum of known genetic disorders

WES is frequently requested after one or more genes deemed relevant to the patient’s clinical presentation had been excluded by Sanger sequencing in hopes of identifying a novel genetic cause. However, many WES studies have highlighted the frequent encounter of disease-causing mutations in known genes that would not have been considered good candidates owing to the marked discrepancy between their published phenotype and the clinical presentation of the patient especially for neurological and dysmorphic disorders, which are often very heterogeneous clinically [19–21]. In a very recent study, for example, we have shown that even in familial cases that are carefully enriched for novel gene discovery by excluding all relevant candidate genes by autozygome analysis, 11 % of WES will reveal mutations in known genes missed by the enrichment step because the presentation was very atypical [22]. Indeed, in many patients with disease-causing mutations identified by the Mendeliome assay, the presentation was sufficiently different from the published phenotype of the respective gene that WES would have been pursued to establish the diagnosis (Table 2). Some of the most dramatic examples are a de novo *EP300* mutation causing microcephalic primordial dwarfism, a homozygous *ZNF526* mutation causing a novel Noonan-like phenotype, a homozygous *IFT122* mutation causing severe ocular anomalies and unusual appendicular skeletal abnormalities, and a de novo *KMT2A* mutation causing genital abnormalities in an affected female, including absent uterus and vagina with remarkable clitoromegaly (Table 2). On the other hand, we have identified mutations in genes typically associated with multisystem disorders in patients with a very limited phenotype, e.g., *BBS4* mutation causing isolated retinal dystrophy instead of Bardet-Biedl syndrome (Table 2). Finally, we note the highly surprising finding of a homozygous nonsense mutation in *TCOF1* causing severe Treacher-Collins syndrome while the carrier parents are completely normal clinically. Interestingly, this mutation had been missed by direct Sanger sequencing of *TCOF1*, most likely because the expectation was a heterozygous peak on the sequence chromatogram given the dominant nature of the disease. This is the first instance of a recessive inheritance of *TCOF1*.

**Table 2:** Summary of clinical features observed in the atypical cases detected by gene panel test^a^

		Mutation					Observed phenotype compared with published phenotype(s)	
Sample ID	Gene name	Type	Status	Origin	Type of panel	Published phenotype(s) related to the case	Typical/previously reported feature(s)	Atypical feature(s)
DG08RC00033	*FGFR2*	Missense	HTZ	De novo	DD	Craniosynostosis syndromes	Craniofacial anomalies	Upper eyelid coloboma
							Sacrococcygeal tail	
		NM_000141.4: c.870G > C (p.Trp290Cys)					Syndactyly	
							Neonatal teeth(with Beare-Stevenson cutis gyrata syndrome)	
							Choanal atresia	
							Thinning of the genu of corpus callosum (with Pfeiffer syndrome)	
11DG0118	*HRAS*	Missense	HTZ	De novo	DD	Costello syndrome	Dysmorphic facies	Corneal haziness
		NM_005343.2: c.34G > A (p.Gly12Ser)					Multiple joint dislocations	Tracheomalacia and bronchomalacia
10DG1851	*COL2A1*	Missense	HTZ	De novo	DD	Spondylometaepiphyseal dysplasia	Disproportionate short stature	Valvular disease (mild TR, MR)
							Thoracic dextroscoliosis	Acanthosis nigricans
							Metaphyseal dysplasia	
		NM_033150.2: c.3023G > T (p.Gly1008Val)					Inguinal hernia	
							Myopia	
09DG01603	*TCOF1*	Nonsense	HMZ	Inherited	DD	Treacher Collins syndrome 1	Underdevelopment zygoma	Confirmed to be inherited as AR
							Choanal atresia	
		NM_001008657.2: c.2656C > T (p.Gln886*)					Microtia	
							No external auditory meatus	
							Malformed ossicles and semicircular canal	
							Iris/optic disc coloboma	
10DG1847	*BRAF*	Missense	HTZ	De novo	DD	Cardiofaciocutaneous syndrome	Hypotonia	Coarse face similar to Costello syndrome
		NM_004333.4: c.1789C > G (p.Leu597Val)					Speech delay	No cardiac defects
								Acanthosis nigricans
								Deep palmar and plantar creases
12DG0359	*EP300*	Nonsense	HTZ	De novo	DD	Rubinstein-Taybi syndrome 2	None	Atypical dysmorphic facies
								Microcephalic primordial dwarfism
		NM_001429.3: c.1092C > A (p.Cys364*)						
11DG2179	*NFIX*	Nonsense	HTZ	Unknown	DD	Sotos syndrome type 2	Overgrowth	Marfanoid habitus
				(Father was not tested)			GDD	Normal bone age
		NM_001271043.2: c.544G > T (p.Glu182*)						
13DG0355	*GNS*	Frameshift	HMZ	Inherited	DD	Mucopolysaccharidosis type IIID	Mild coarse face	Advanced RP
		NM_002076.3: c.83del (p.Leu28Argfs*37)					Mild hepatomegaly	
							Clear cornea	
							Skeletal manifestations	
11DG2478	*COL11A2*	Missense	HMZ	Inherited	DD	Otospondylomegaepiphyseal dysplasia	Epiphyseal dysplasia	Hypoplastic optic nerve
		NM_001163771.1: c.654 T > A (p.Tyr218*)						Mitral valve prolapse and regurgitation
							Deafness	
14DG1212	*IFT122*	Splice site	HMZ	Inherited	DD	Cranioectodermal dysplasia 1	Nystagmus	Iris and optic nerve coloboma
							Metaphyseal dysplasia	Microphthalmia
		NM_052990.2:c.2042 + 2 T > C						Duplicated thumb and big toe
								Post-axial polydactyly
								Very short tibiae compared with fibulae
13DG2208	*ROR2*	Missense	HMZ	Parents not tested	DD	Robinow syndrome	Vertebral anomalies(in Robinow syndrome)	Atypical fibrochondrogenesis-like skeletal dysplasia
		NM_004560.3: c.1970G > A (p.Arg657His)				Brachydactyly, type B1		
10DG0298	*KMT2A*	Frameshift	HTZ	De Novo	DD	Wiedemann-Steiner syndrome	Dysmorphic facies	Absent uterus and vagina, remarkable clitoromegaly
		NM_001197104.1: c.7567_7570del (p.Val2523Lysfs*2)						
08DG-00226	*NSD1*	Nonsense	HTZ	De Novo	DD	Sotos Syndrome 1	Dysmorphic facies	No overgrowth
		NM_022455.4: c.2058 T > A (p.Tyr686*)					Thin corpus callosum, PVL and colpocephaly on MRI brain	
10DG0615	*CNNM4*	Missense	HMZ	Parents not tested	Vision	Jalili syndrome	LCA, retinal degeneration	Retinal coloboma
		NM_020184.3: c.734C > T (p.Ser245Leu)						No dental anomalies
12DG0398	*BBS4*	Frameshift	Compound HTZ	Parents not tested	Vision	Bardet-Biedl syndrome 4	-RP	Lack of other features of BBS
		NM_001252678.1:c.795_796insT(p.Lys266*)						(isolated RP)
		NM_033028.4:c.262delG (p.Glu88Asnfs*54)						
14DG0073	*ATRX*	Nonsense	Hemizygous	Inherited	Neuro	Mental retardation-hypotonic facies syndrome, X-linked	Microcephaly	RP
		NM_000489.3:c.7156C > T (p.Arg2386*)					GDD	Optic disc coloboma
							White matter changes	
12DG1296	*ALMS1*	Frameshift	HMZ	Inherited	Vision	Alstrom syndrome	Achromatopsia	Lack of other features of Alstrom syndrome (isolated achromatopsia)
		NM_015120.4: c.1674delT (p.Pro559Leufs*37)						
12DG1554	*STXBP1*	Missense	HTZ	De novo	Neuro	Epileptic encephalopathy, early infantile, 4	Seizures	Pigmentary retinal changes
		NM_001032221.3: c.874C > T (p.Arg292Cys)						
13DG1051	*CDKL5*	Nonsense	HMZ	Inherited	Neuro	Epileptic encephalopathy, early infantile, 2	GDD	Macrocephaly and overgrowth
		NM_001037343.1: c.2854C > T (p.Arg952*)						Facial dysmorphism similar to Sotos syndrome but normal bone age and negative *NSD1* mutation
								No seizures or regression
								No abnormal movements
10DG2024	*GTDC2*	Missense	HMZ	Inherited	Neuro	Muscular dystrophy-dystroglycanopathy (congenital with brain and eye anomalies, type A, 8)	None	Isolated large occipital encephalocele
	*(POMGNT2)*							No polydactyly
		NM_032806.5: c.473G > A (p.Arg158His)						Neonatal death
12DG0133	*HSD17B4*	Missense	HMZ	Inherited	Neuro	D-bifunctional protein deficiency	Neonatal seizures	Normal brain MRI
								No skeletal manifestations or stippling
		NM_001199291.1: c.442C > G (p.His148Asp)						No eye findings
								No dysmorphism
10DG0415	*ATN1*	Missense	HTZ	De novo	Neuro	Dentatorubro-pallidoluysian	None	Early onset static encephalopathy
		NM_001940.3: c.3178C > T (p.His1060Tyr)				atrophy		Novel molecular mechanism (point mutation)
12DG0705	*KIAA0196*	Missense	HTZ	De Novo	Neuro	Ritscher-Schinzel syndrome	Seizures	Normal brain MRI
		NM_014846.3: c.1669G > A (p.Ala557Thr)				Spastic paraplegia 8, AD	Speech delay and learning disability	No ataxia or spasticity
10DG0351	*ADRA2B*	Nonsense	HMZ	Inherited	Neuro	Non-syndromic ID	None	Microcephaly
		NM_000682.6: c.664C > T (p.Arg222*)						GDD
12DG0532	*ZNF526*	Missense	HMZ	Inherited	Neuro	Mild non-syndromic ID	None	Novel Noonan like phenotype
								GDD
		NM_133444.1: c.479A > C (p.Lys160Thr)						
14DG0745	*WDR45B*	Nonsense	HMZ	Inherited	Neuro	ID and microcephaly	Primary microcephaly	Epilepsy
	*(*WDR45L)	NM_019613.3: c.673C > T (p.Arg225*)						White matter changes, brain atrophy, hypoplastic corpus callosum
13DG2156	WDR81	Nonsense	HMZ	Inherited	Neuro	Cerebellar ataxia, mental retardation, and dysequilibrium syndrome 2	Cerebellar hypoplasia	Normal corpus callosum
		NM_152348.3: c.133C > T (p.Gln45*)						Prenatal onset complicated by neonatal death

^a^Atypical case is defined as a case that has unusual clinical features, unusual mode of inheritance, a novel phenotype or lack of typical features

*AD,* autosomal dominant, *AR*, *BBS* Bardet-Biedl syndrome, *DD* Dysmorphia-Dysplasia panel, *FTT* failure to thrive, *GDD* global developmental delay, *HMZ* Homozygous, *HTZ* Heterozygous, *ID* intellectual disability, *LCA,* Leber congenital amaurosis, *MR*, *MRI* magnetic resonance imaging, *PVL,* Periventricular leukomalacia, *RP* retinitis pigmentosa, *TR,* Tricuspid regurgitation

### Improved annotation of the human variome

Large scale genomic studies offer opportunities to improve the annotation of the human variome. This study, in which more than 2300 well phenotyped human patients in a highly consanguineous population have been specifically tested for established disease genes, offers several advantages. First, we were able to confirm genes that were only considered candidates because their candidacy was based on single mutations/families so their status based on this study should be upgraded in OMIM as such (e.g., *ARL14EP*, *ZNF526*, *WDR45B*, and *WDR81*). Second, we have added 433 novel disease alleles from a total of 788 variants, the largest to be reported in a single study (Additional files 1 and 2). Interestingly, 22 variants were concurrently added to the Human Gene Mutation Database (HGMD) after submission of the manuscript. These are in the HGMD release of 2015 and not in that of 2014. Third, the very large number of variants we identified in the course of this study represent an unprecedented resource on the Arab variome (nearly all patients in this study were Arab in ethnicity), and this will be invaluable to the interpretation of clinical molecular genetic tests on Mendelian genes in Arab patients since it will help address the uncertainty surrounding the identification of many Arab-specific or Arab-enriched variants (Additional file 1). Fourth, the high degree of consanguinity allowed us to observe many variants in homozygosity as a result of autozygosity. This is particularly helpful when these variants were previously reported as disease-causing because observing them in the homozygous state at a relatively high population frequency strongly argues against their purported disease link. Additional file 3 includes 342 HGMD variants that appeared at high frequency (minor allele frequency [MAF] >1 %) in our in-house database, including 133 variants with MAF >5 %. Of these variants with MAF >1 % in our cohort, 137 are listed in the 1000 Genomes Project with a MAF <1 %, highlighting the unique distribution of variants in our population and the value it adds to the annotation of the human variome. Furthermore, our finding of previously reported disease genes that harbor apparently inactivating mutations in the homozygous state at a relatively high frequency and in patients who lack the purported phenotype challenges their listing as disease genes (e.g., *CACNA1F*, *MYH8*, and *PRX1*) although we acknowledge a potential role of such confounding factors as reduced penetrance.

## Discussion

Genomics have ushered in a new era for clinical medicine [23]. The ability to scan the entire genome (or its coding part) for disease-causing mutations relatively free of clinical bias has uncovered the limited sensitivity and specificity of making diagnoses on clinical grounds only. This was first apparent with the advent of array comparative genomic hybridization, which specifically targets large genomic mutations. Subsequently, whole genome sequencing (WGS) and WES confirmed the same pattern. This raises the interesting question of whether all patients with a suspected genetic diagnosis should have WGS/WES as the initial diagnostic test [24]. Pending data on the validity of this approach, one has to consider some practical challenges. Cost remains a significant hurdle that prevents most patients, especially in less wealthy countries, from accessing WGS/WES. While the running cost will continue to decrease, the challenge of identifying a single causal variant from among tens of thousands will remain formidable for the foreseeable future [25]. In addition, debate still rages over the issue of incidental findings, with changing guidelines reflecting the strong and sound arguments made by camps on either side of the debate, especially in pediatrics [26–28]. Gene panels that specifically target a disease relevant to the patient’s presentation appear to address some of these limitations but suffer from lack of uniformity in design and are typically too focused on a particular phenotype such that they may miss atypical presentations.

In this study, we sought to develop an assay that addresses the above-mentioned limitations. In the design stage, we limited ourselves to genes that are very likely to be disease-causing in a Mendelian context based on the best available evidence in order to eliminate the uncertainty surrounding the finding of variants in genes not known to be linked to human diseases. We mainly included genes whose pathogenicity was supported by the presence of two pathogenic alleles. However, exceptions were made for genes with a single reported mutation but are further supported by compelling mouse data or positional mapping data. This is important because it has to be acknowledged that clinical WGS/WES currently appears to saddle the divide between clinical care and research [29]. If the Mendeliome assay is negative, it may be easier to prepare the patient for the possibility of identifying a novel genetic cause by WGS/WES that requires confirmation in a research setting. Unlike currently available gene panels, we sought to be as inclusive as possible to minimize the challenge of atypical cases. For example, a gene for myopia presenting with ectopia lentis would still be identified because virtually every gene known to present with a prominent eye phenotype was included in the Vision panel [30]. Indeed, our analysis showed that only 3 % (62/2,357) of cases may have been missed because the gene was not included in the right panel and even this limitation can be addressed through the spike-in design. Such broad and inclusive design was particularly helpful in disease categories that are characterized by very high rates of heterogeneity. In addition to the Vision panel, we also note the high rate of atypical cases identified by the Dysmorphology/Dysplasia, Neurology and Immunology panels, although such cases were encountered in nearly all the panels.

## Conclusions

Our design brought about a dramatic reduction in cost both in terms of reagents and interpretation. Although clinical WES/WGS with exceptionally rapid turnaround time has been reported, those were exceptional scenarios that required a level of infrastructure and logistics well beyond a typical healthcare facility [31]. On the other hand, our turnaround time (one week from receiving the sample to calling the candidate variants and three days to confirm the variants by Sanger) can be offered routinely as a service to any healthcare provider. Therefore, our test can be viewed as a potential first tier test that is relatively inexpensive and rapid with a straightforward consenting process. If negative, a more comprehensive genomic test can be ordered as a second-tier test such that the higher order complexities (cost, consent, etc.) associated with this test can be confined to a smaller number of individuals than it would otherwise be if it were offered as a first-tier test.

## Materials and methods

### Human subjects

All families were enrolled under several King Faisal Specialist Hospital and Research Centre institutional review board-approved protocols (depending on the phenotype), after signing a written informed consent. These included: “Genetics of vision impairment in Saudi Arabia” (RAC# 2070 023 Research Advisory Council), “Genetics of craniofacial birth defects” (RAC# 2080 006), “Characterization of peroxisomal biogenesis disorders in Saudi Arabia” (RAC# 2080 033), “Genetics of OI in Saudi Arabia” (RAC# 2090 035), and “Study of Mendelian phenocopies of common diseases” (RAC# 2121 053). The study was carried out in accordance with the declaration of Helsinki.

### Defining the Mendeliome

Patients with various hereditary disorders most often are referred to the medical geneticist either through their primary care provider or through a medical subspecialist who attended to the most prominent clinical presentation (i.e., neurological, ophthalmology, skin, renal, hematological, etc.). Therefore, we sought to design our symptom/sign-based gene panels, collectively known as the “Mendeliome” in a way that simulates the way these patients present in clinical practice to the respective specialty. Mendelian disorders are defined as hereditary disorders caused by a single autosomal or X-linked gene. The OMIM database, which currently contains about 4300 monogenic disorders associated with known molecular defects, represents the most comprehensive source of such information on monogenic disorders. Therefore, it was used as the primary source for gene identification. However, it was manually curated to ensure that only genes with confirmed links to disease are included. It was also supplemented with additional data from PubMed, the Genetic Testing Registry, and Gene Tests. As such, 13 gene panels which cover the spectrum of “pediatric and adult” clinical genetic medicine were constructed. Within each panel, genes were sorted based on the most prominent sign/symptom with which they are most likely to be associated upon presentation to clinical care. This presentation may help the referring clinician, and without requiring sophisticated knowledge about these genes, decide on the appropriateness of genetic testing using these gene panels. Since many genetic disorders are as likely to present to several medical specialties, we allowed for redundancy between the different panels (average 15 %) such that a gene may be present in more than one panel.

### Primer design and multiplexing assay development

We used 3070 genes covering over 4,000 Mendelian disorders as annotated by OMIM up to August 2013 as a basis for the design and synthesis of highly multiplexed gene panels using Ion AmpliSeq Designer software (Life Technologies, Carlsbad, CA, USA). Additional file 4 displays the list of genes, their corresponding panels, information about the used transcripts, physical positions, and number of exons. Of these 3070 genes, 2826 are already listed in the Genetic Testing Registry. Thirteen panels encompassing nearly all of the OMIM genes were defined broadly based on clinical disciplines with some redundancy in gene content of individual panels. Primer design was based on generating amplicons with an average length of 200 bp providing 90 % minimum coverage of the coding DNA sequence, and, on average, 10 bp flanking regions of associated exons. Additional file 5 contains all AmpliSeq primers used in the assay. Following in silico design, coverage was assessed for compliance with design criteria and manual processes applied on a gene by gene basis to ensure adequate coverage and resolve factors such as 3’ SNPs that could impact primer efficiency. Primers for each panel were then synthesized and pooled into two multiplex reactions based on PCR compatibility minimizing likelihood of primer–primer interactions. Following synthesis, primer pools were tested for coverage, recommended multiplexing and other quality control metrics to ensure specifications were met. Panels ranged from 96–758 genes with >90 % coverage in 97–100 % of genes in each panel. Additional file 6 shows information about the different panels and related design information.

### Library preparation and NGS

DNA samples (10 ng of each) were treated to obtain the Ion Proton AmpliSeq library for one of the 13 gene panels as appropriate. DNA was amplified using gene panel Primer Pools, AmpliSeq HiFi mix (Thermo Fisher, Carlsbad, CA, USA) and 10–15 amplification cycles. PCR pools for each sample were combined and subjected to primer digestion with FuPa reagent (Thermo Fisher, Carlsbad, CA, USA). Pooled amplicons were then ligated with universal adapters. After purification, libraries were quantified by quantitative PCR and normalized to 100 pM. Normalized libraries were barcoded (ligated with 24 different Ion Xpress Brcode adapters) and pooled in equal ratios for emulsion PCR (ePCR) on an Ion OneTouch System. Following ePCR, templated Ion Sphere particles were enriched using the Ion OneTouch ES enrichment system enrichment system. Both ePCR and enrichment procedures followed the manufacturer’s instructions (Thermo Fisher, Carlsbad, CA, USA). The template-positive Ion PI Ion Sphere particles were processed for sequencing on the Ion Proton instrument (Thermo Fisher, Carlsbad, CA, USA).

### Data processing and bioinformatics analysis

The data of each run were analyzed through a multistep pipeline. Additional file 7 summarizes the analysis workflow (pipeline), and the details of the variant filtration sub-pipeline. The left side of Additional file 7 demonstrates the basic steps of our variant detection workflow. In the first step of this pipeline, the quality of the reads is verified and regions of the reads with low quality (less than 20) are trimmed out before alignment. The runs with low yield after this quality check are excluded. Additional file 8 shows the quality data for the runs per panel at both Q0 and Q17 (results per panel is the average for all samples in the panel). In the second step, the reads are aligned to the reference hg19 sequence. The alignment program was tmap, which is distributed within the Ion Torrent Suite (Thermo Fisher, Carlsbad, CA, USA) [32]. Additional file 9 shows statistics about the alignment and coverage related to the target regions as well as the observed depth per panel (results per panel is the average for all samples in the panel). The observed depth after alignment ranges from 162× (for the Neurology panel including 758 genes) to 840× (for the Renal panel including 96 genes). It can be observed from Additional files 8 and 9 that the data are of good quality and the observed depth is very close to the expected depth. We can also conclude that it is possible to increase the number of samples multiplexed per chip for panels with low numbers of genes, such as the Renal and Pulmonary panels, which reduces the cost without affecting the quality. In the third step, the aligned reads were processed for variant calling using the Torrent Suite Variant Caller (TVC) program, which is tuned to Ion Torrent data and considers quality scores and manufacturer-specific details. In the subsequent step, the variants are annotated using public knowledge databases as well as in-house variant databases, using in-house programs that extend the public Annovar package. The public databases include those available from the Annovar package and other commercial datasets like HGMD [33]. The in-house databases include collections of disease-causing variants published by different Saudi teams and aggregation of the variants produced by the samples in this paper. In the final step of the pipeline, the non-relevant variants are filtered out based on their functional characteristics and their abundance in our datasets. The right side of Additional file 7 illustrates the details of the variant filtering process. Variants that are less likely to play a functional role (intronic and synonymous) and variants that were present in population databases (e.g., in the 1000 Genomes Project database with MAF >1 %) are filtered out. Furthermore, variants that are frequent in our in-house database are also filtered out; a variant with more than 20 occurrences were considered frequent. (The cutoff of 20 occurrences has been selected on test data to assure 100 % sensitivity.) An individual base quality of 100 (using Phred-like score produced by the Torrent Suite base caller) is also selected to exclude low confident variants. The few remaining variants are then analyzed based on relevance of gene to phenotype, zygosity (when indicated), and SIFT and PolyPhen scores (for missense variants). Table 3 shows the efficiency of our filtering strategy. It shows that the subsequent filtering steps lead to a short list of variants to be examined by domain experts. As the tables show and as expected, the larger the panel, the larger the list. It is also important to note that having more samples included in the in-house database leads to more filtration power and makes the list even shorter. Ultimately, the recognized causal variant is identified as pathogenic or likely pathogenic as defined by the recent American College of Medical Genetics and Genomics (ACMG) guidelines (“Standards and guidelines for the interpretation of sequence variants: a joint consensus recommendation of the American College of Medical Genetics and Genomics and the Association for Molecular Pathology”), and the extensive variant data obtained by sequencing thousands of ethnically comparable patients (Saudis) was helpful in applying population frequency as a reliable criterion for pathogenicity in this study.

**Table 3:** The results of applying filtering steps over all the variant files (including those with no relevant variant detected)

Panel	Input	Functional sites	Public Pop. DBs	SGP Pop. DB	Quality	Zygosity
Cardiovascular	746	338	76	26	9	2
Deafness	828	257	63	50	17	6
Dermatology	1113	271	71	41	17	5
Dysmorphology/Dysplasia	1529	369	80	43	15	2
Endocrinology	1129	326	61	42	19	5
Gastroenterology	362	190	60	20	6	1
Hematology	1474	324	79	39	18	3
Inborn Errors of Metabolism	1955	571	94	54	24	4
Neurology	2885	718	158	87	29	4
PID	633	309	111	22	6	1
Pulmonology	723	230	74	39	21	3
Renal	507	132	35	21	7	1
Vision	906	341	75	51	17	3
Total (averages)	1138	337	80	41	16	3

The table entries are the average number of variants per panel after applying the respective filter in the column title. The column titled ‘Input’ is the input without applying any filter. The column titled ‘Functional sites’ is for the step of filtering intronic, UTR, synonymous variants. The column titled ‘Public Pop. DBs’ is for the step of filtering variants based on the 1000 Genomes Project database. The column titled ‘SGP Pop. DBs’ is for the step of filtering variants based on the in-house database. The column titled ‘Quality’ is for the step of filtering based on the quality criteria. The column titled ‘Zygosity’ is for the step of excluding non-homozygous variants

#### Molecular karyotyping

Given that the Mendeliome assay is inherently limited to established disease genes and will miss cases caused by large structural variants, we randomly selected 213 cases that are negative by the Mendeliome assay and processed them using molecular karyotyping. The CytoScan HD (Affymetrix) array was used for the majority of our patients. This array platform contains 2.6 million markers for CNV detection (Affymetrix), of which 750,000 are genotype SNPs and 1.9 million are nonpolymorphic probes, for the whole genome coverage. The analysis was performed using the Chromosome Analysis Suite version Cyto 2.0.0.195(r5758). Oligonucleotide probe information is based on build 37 of the UCSC Genome Browser (GRCh37/hg19) [34].

Briefly, 250 ng of genomic DNA was digested with the restriction enzyme NspI and then ligated to an adapter, followed by PCR amplification using a single pair of primers that recognized the adapter sequence. The PCR products were run on a 2 % Tris-borate-EDTA (TBE) gel to confirm that the majority of products were between 150 and 2000 bp in length. To obtain a sufficient quantity of PCR product for further analysis, all products from each sample were combined and purified using magnetic beads (Agencourt AMPure, Beckman Coulter, Beverly, MA, USA). The purified PCR products were fragmented using DNase I and visualized on a 4 % TBE agarose gel to confirm that the fragment sizes ranged from 25 to 125 bp. The fragmented PCR products were subsequently end-labeled with biotin and hybridized to the array. Arrays were then washed and stained using a GeneChip Fluidics Station 450 and scanned using an Affymetrix GeneChip Scanner 3000 7G. Scanned data files were generated using Affymetrix GeneChip Command Console Software (version 1.2) and analyzed with the Chromosome Analysis Suite.

The hidden Markov model available within the Chromosome Analysis Suite software package was used to determine the copy-number states and their breakpoints. Thresholds of log2 ratio ≥0.58 and ≤ −1 were used to categorize altered regions as CNV gains (amplification) and copy number losses (deletions), respectively.

To minimize the detection of false-positive CNVs arising due to inherent microarray “noise”, only alterations that involved at least 50 consecutive probes and that were at least 500 kb in size were used to categorize altered regions as CNV gains (amplification), whereas those at least 200 kb in size were used to categorize copy number losses (deletions).

We then proceeded to evaluate the CNVs detected in our patients based on the ACMG standards and guidelines. The genic content in the CNV interval of all the patients who had a molecular karyotype performed was taken into consideration by seeking recent publications to compare breakpoints, phenotypes, and different sizes of CNVs that overlapped. To exclude aberrations representing common benign CNVs, all the identified CNVs were compared with those reported in the Database of Genomic Variants [35] and those reported in our own database for individuals who have been classified as normal.

De novo CNVs that met the size cutoff of 200 kb for deletions and 500 kb for duplications (based on the laboratory’s consideration of the performance characteristics of the assay used) and were not found in either parent were classified as pathogenic. However, this does not eliminate the possibility that pathogenic CNVs exhibiting incomplete penetrance or variable expressivity can be present in an unaffected parent.

#### Whole exome sequencing and analysis

The remaining 178 were processed using WES. Each DNA sample (100 ng) was treated to obtain the Ion Proton AmpliSeq library. Briefly, DNA was amplified in 12 separate wells using Exome Primer Pools, AmpliSeq HiFi mix (Thermo Fisher, Carlsbad, CA, USA) and ten amplification cycles. All 12 PCR pools were combined in one well and subjected to primer digestion performing incubation with FuPa reagent (Thermo Fisher, Carlsbad, CA, USA). Amplified exome targets were ligated with Ion P1 and Ion Xpress Barcode adapters. After purification libraries were quantified using quantitative PCR with the Ion Library Quantification Kit (Thermo Fisher, Carlsbad, CA, USA). The prepared exome library was further used for emulsion PCR on an Ion OneTouch System and templated Ion Sphere particles were enriched using Ion OneTouch ES, both procedures following the manufacturer’s instructions (Thermo Fisher, Carlsbad, CA, USA). The template-positive Ion PI Ion Sphere particles were processed for sequencing on the Ion Proton instrument (Thermo Fisher, Carlsbad, CA, USA). Approximately 15–17 Gb of sequence was generated per sequencing run. Reads were mapped to UCSC hg19 [36] and variants identified using the Saudi Human Genome Program (SHGP) pipeline.

### Availability of data

All the variants generated on the participants of this study, including disease and non-disease causing mutations from the gene panels and from WES data, are accessible through the Saudi Variome Database [37, 38]. Tabulated versions of the data are provided in Additional files 1, 2, 3, 4, 5, 6, 8, and 9 and can be accessed through [37, 38]. The exome files can be accessed through [37, 38]. The ClinVar accession numbers for the novel variants in this paper are SCV000221340–SCV000221740.

## Additional files

Additional file 1: Table S1. List of unique variants that are disease-causing. Additional file 2: Table S3. List of novel variants. Additional file 3: Table S2. Frequent variants encountered in our study that are designated as disease-mutation by HGMD. This table includes 342 HGMD variants that appeared at high frequency (MAF >1 %) in our in-house database. Of these, 133 variants appeared at MAF >5 %. We also observe that the MAF of 137 of them in the 1000 Genomes database is less than 1 %. We also compared the HGMD release of 2013 with that of 2014. We found that in the 2014 release of HGMD, there are 16 new variants with MAF >5 %. Additional file 4: Table S7. List of genes included in the Mendeliome assay. Additional file 5: Table S8. Gene panel primers – contains all AmpliSeq primers used in the assay. Additional file 6: Table S4. Gene panel design information. Additional file 7: Figure S1. Variant detection and analysis workflow. On the left side we show the basic steps of our variant detection workflow. The base calling step involves the transformation of signal data into base space (A/C/G/T). The output of this step is an unaligned reads in BAM format. The mapping is the process of aligning the reads to the reference human genome (hg19). The output of this step is alignments of all reads (BAM format). The variant calling involves the detection of variations in the aligned reads. The variant filtration step involves the exclusion of variants not related to the disease. On the right side, we show the details of the variant filtration workflow. The first step involves the exclusion of deep intronic variants (more than 20 bases far from the exon terminals), UTRs, and non-frameshift indels. Then variants that are frequent in public databases (the 1000 Genomes database) with MAF >1 % are excluded. Variants that are also frequent in our in-house database are also excluded (MAF >1 %). The remaining variants are then filtered based on a score (>100) computed by Torrent Suite according to different criteria like confidence of base calls, depth, and context bases. The final step involves filtering based on zygosity (if the variant is homozygous). Then domain experts evaluate the variants based on their knowledge about the phenotype. The remaining short list of variants (if not empty) is then sent to the Sanger team to validate the variants. The numbers next to each step in the filtration is the average number of the remaining variants after applying the next filtering step. Additional file 8: Table S5. Sequencing quality data for the runs per panel (entries are the average of all runs per panel). Additional file 9: Table S6. Read alignment quality per panel (entries are the average of all runs per panel).

## Abbreviations

ACMG: American College of Medical Genetics and Genomics
bp: base pair
CNV: copy number variation
ePCR: emulsion PCR
HGMD: Human Gene Mutation Database
MAF: minor allele frequency
NGS: next-generation sequencing
OMIM: Online Mendelian Inheritance in Man
PCR: polymerase chain reaction
SNP: single nucleotide polymorphism
TBE: Tris-borate-EDTA
UTR: untranslated region
WES: whole exome sequencing
WGS: whole genome sequencing
