# Erratum to: Comprehensive gene panels provide advantages over clinical exome sequencing for Mendelian diseases

**DOI:** 10.1186/s13059-015-0798-7

**Published:** 2015-10-13

**Authors:** Saudi Mendeliome Group

**Affiliations:** Saudi Human Genome Project, King Abdulaziz City for Science and Technology, Riyadh, Saudi Arabia

## Erratum

Unfortunately, the original version of this article [1] contained a mistake. In the Acknowledgement section of the article the author Saeed Bohlega was incorrectly spelt as Saeed Boholega. We apologise for this error. The author’s name has been included correctly in the list below:

## Acknowledgement

This study was supported by a research grant from King Salman Center for Disability Research (FSA) as well as the Saudi Human Genome Project initiative, KACST.Saudi Mendeliome Group (alphabetical order): Abdulwahab, Firdous; Abouelhoda, Mohamed; Abouthuraya, Rula; Abumansour, Iman; Ahmed, Syed O.; Al Rubeaan, Khalid; Al Tassan, Nada; AlAbdulaziz, Basma; AlAbdulrahman, Khalid; Alamer, FH; Alazami, Anas; Al-Baik, Lina A.; Aldahmesh, Mohammed; Al-Dhekri, Hasan; AlDusery, Haya; Algazlan, Sulaiman; Al-Ghonaium, Abdulaziz; Alhamed, Mohammed; Alhashem, Amal; Alhissi, Safa Ahmed; AlIssa, Abdulelah; Aljurf, Mahmoud D.; Alkuraya, Fowzan S; Alkuraya, Hisham; Allam, Rabab; Almasharawi, Iman J; Almoisheer, Agaadir; AlMostafa, Abeer; Al-Mousa, Hamoud; Al-Muhsen, Saleh; Almutairy, Eid A; Alnader, Noukha; AlNaqeb, Dhekra; ALOtaibi, AB; Alotibi, Afaf; Al-Qattan, Sarah; Al-Saud, Bandar; Al-Saud, Haya; Alshammari, M; Alsharif, Hadeel; Alsheikh, Abdulmoneem H; Al-Sulaiman, Ayman; Altamimi, AS; Al-Tayeb, Hamsa; Alwadaee, SM; Al-Younes, B; Alzahrani, Fatima; Anazi, Shamsa; Arnaout, Rand; Bashiri, Fahad; Binamer, Yousef; Binhumaid, FS; Bohlega, Saeed; Broering, Dieter; Burdelski, Martin; Dasouki, Majed Jamil; Dzimiri, Nduna Francis; Elamin, Tanziel; El-Kalioby, Mohamed; Elsiesy, Hussien; Faqeih, Eissa; Faquih, Tariq; Hagos, Samya; Hagr, Abdulrahman A; Hashem, Mais; Hawwari, Abbas; Hazzaa, Selwa; Ibrahim, Niema; Imtiaz, Faiqa; Jabr, Amal; Kattan, Rana; Kaya, Namik; Kentab, Amal; Khalil, Dania; Khan, Arif O; Khier, Omnia; Meyer, Brian; Mohamed, Jawahir; Monies, Dorota; Muiya, Paul N.; Murad, Hatem; Naim, EA; Owaidah, Tarek; Patel, Nisha; Ramzan, Khushnooda; Salih, Mustafa A; Shagrani, Mohammad; Shaheen, Ranad; Shamseldin, Hanan; Sogaty, Sameera; Subhani, Shazia; Taibah, Khalid; Wakil, Salma M.
