# Supplementary material for: Comprehensive gene panels provide advantages over clinical exome sequencing for Mendelian diseases
Source: Genome Biol. 2015 Jun 26;16(1):134. doi: 10.1186/s13059-015-0693-2 (PMC4499193; doi:10.1186/s13059-015-0693-2)
Supplement: Additional file 7: Figure S1. — Variant detection and analysis workflow. On the left side we show the basic steps of our variant detection workflow. The base calling step involves the transformation of signal data into base space (A/C/G/T). The output of this step is an unaligned reads in BAM format. The mapping is the process of aligning the reads to the reference human genome (hg19). The output of this step is alignments of all reads (BAM format). The variant calling involves the detection of variations in the aligned reads. The variant filtration step involves the exclusion of variants not related to the disease. On the right side, we show the details of the variant filtration workflow. The first step involves the exclusion of deep intronic variants (more than 20 bases far from the exon terminals), UTRs, and non-frameshift indels. Then variants that are frequent in public databases (the 1000 Genomes database) with MAF >1 % are excluded. Variants that are also frequent in our in-house database are also excluded (MAF >1 %). The remaining variants are then filtered based on a score (>100) computed by Torrent Suite according to different criteria like confidence of base calls, depth, and context bases. The final step involves filtering based on zygosity (if the variant is homozygous). Then domain experts evaluate the variants based on their knowledge about the phenotype. The remaining short list of variants (if not empty) is then sent to the Sanger team to validate the variants. The numbers next to each step in the filtration is the average number of the remaining variants after applying the next filtering step. [file 13059_2015_693_MOESM7_ESM.ppt]

## Slide 1
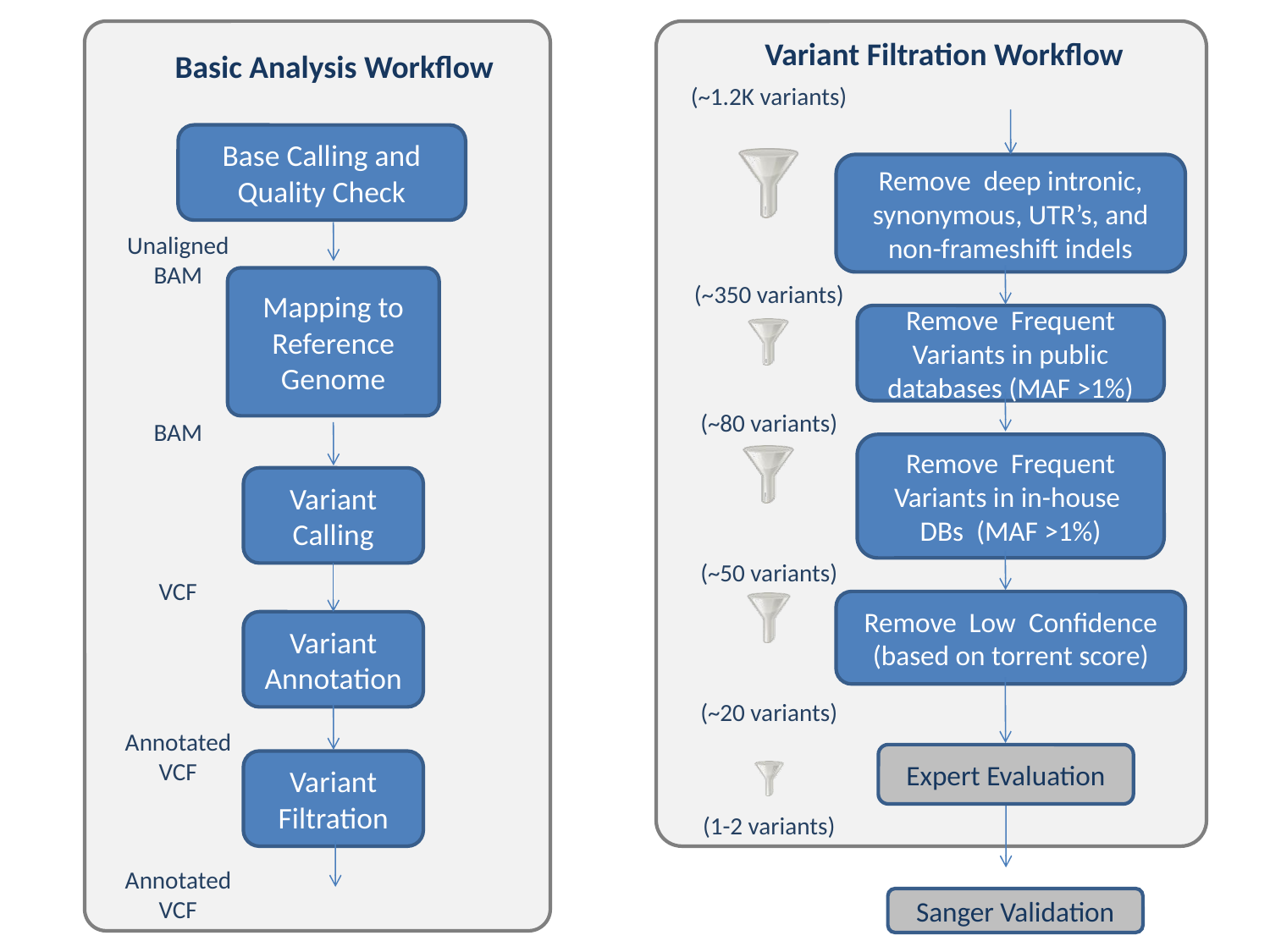

Variant Filtration Workflow
Ba
Basic Analysis Workflow
(~1.2K variants)
Base Calling and Quality Check
Remove deep intronic, synonymous, UTR’s, and non-frameshift indels
Unaligned BAM
Mapping to Reference Genome
(~350 variants)
Remove Frequent Variants in public databases (MAF >1%)
(~80 variants)
BAM
Remove Frequent Variants in in-house DBs (MAF >1%)
Variant Calling
(~50 variants)
VCF
Remove Low Confidence (based on torrent score)
Variant Annotation
(~20 variants)
Annotated VCF
Expert Evaluation
Variant Filtration
(1-2 variants)
Annotated VCF
Sanger Validation
